# Effects of Different Seedless Treatments on Fruit Quality and Coloring of ‘Jumeigui’ Grapes

**DOI:** 10.3390/plants15050742

**Published:** 2026-02-28

**Authors:** Dawei Cheng, Shasha He, Ting Ye, Kejing Zhang, Xiaoxu Sun, Hong Gu, Xiangyang Tong, Ming Li, Lan Li, Jinyong Chen

**Affiliations:** 1 Zhengzhou Fruit Research Institute, Chinese Academy of Agricultural Sciences, National Key Laboratory for Germplasm Innovation & Utilization of Horticultural Crops, Zhengzhou 450009, China; chengdawei@caas.cn (D.C.); heshasha1213@163.com (S.H.);; 2 Zhongyuan Research Center, Chinese Academy of Agricultural Sciences, Xinxiang 453500, China; 3 College of Horticulture and Plant Protection, Henan University of Science and Technology, Luoyang 471000, China

**Keywords:** ‘Jumeigui’ grape, forchlorfenuron, thidiazuron, 6-benzyladenine, fruit quality

## Abstract

To investigate the effects of different seedless treatments on grape coloring and fruit quality, *Vitis vinifera* × *Vitis labrusca* cv. ‘Jumeigui’ were treated with different concentrations of forchlorfenuron (CPPU) (0.5, 1 and 1.5 mg/L), thidiazuron (TDZ) (0.5, 1 and 1.5 mg/L), and 6-benzyladenine (6-BA) (10, 20 and 30 mg/L) in combination with 18 mg/L gibberellic acid (GA_3_) during the seedless-fruit-setting period. After the grapes ripened, multiple quality indicators were measured to analyze and evaluate the effects of different treatments on the fruit coloration and quality of ‘Jumeigui’ grapes. The results showed that increasing concentrations of CPPU and TDZ gradually reduced the comprehensive fruit quality of ‘Jumeigui’ grapes. The treatments with 18 mg/L GA_3_ + 0.5 mg/L CPPU/TDZ were relatively effective in improving the comprehensive quality of ‘Jumeigui’ grapes. With increasing concentrations of 6-BA, the comprehensive effect initially increased and then decreased. The treatment with 18 mg/L GA_3_ + 20 mg/L 6-BA resulted in a soluble solids content of 20.03% and a coloring index of 4.10, demonstrating the best overall improvement in the comprehensive quality of ‘Jumeigui’ grapes. Based on practical production considerations, it is recommended to apply 18 mg/L GA_3_ + 20 mg/L 6-BA during the seedless-fruit-setting period of ‘Jumeigui’ grapes to enhance coloring effects and improve fruit quality.

## 1. Introduction

The global market for high-quality table grapes exhibits a strong preference for seedless varieties due to their superior eating convenience [1]. To meet this demand, modern viticulture frequently relies on the application of plant growth regulators (PGRs) to induce seedlessness in grape berries—a process in which early embryo abortion results in commercial seedless berries [2]. Among various PGRs, gibberellic acid (GA_3_) serves as a fundamental agent for promoting fruit set and berry enlargement in numerous grape cultivars [3,4,5]. However, the efficacy of GA_3_ alone can be limited, particularly for improving berry retention and achieving optimal fruit size and quality. Therefore, combining GA_3_ with synthetic cytokinins has become a common practice to enhance the comprehensive outcomes of seedless-berry production.

Commonly used cytokinins in grape production include forchlorfenuron (CPPU), thidiazuron (TDZ), and 6-benzylaminopurine (6-BA). These compounds exhibit distinct physiological activities. CPPU and TDZ are highly potent cytokinins that promote cell division, thereby significantly increasing berry size and bunch weight [6,7,8,9,10,11,12,13,14,15]. For instance, treatments with GA_3_ and CPPU have been shown to markedly increase the single-berry weight and bunch weight of grapes [6,7,8,9,10]. Similarly, applications of GA_3_ and TDZ can enhance bunch weight, berry weight and fruit diameter in seedless cultivars [12,13,14,15]. However, their use may also be associated with potential side effects, such as a decrease in soluble solids content (SSC) to some degree, delayed skin coloration, or altered fruit texture [12]. In contrast, 6-BA is generally considered a milder cytokinin, and its co-application with GA_3_ has also been proven effective in promoting grape berry growth and improving fruit quality [16,17]. The selection of the appropriate cytokinin type and concentration is highly genotype-dependent, as the response varies significantly among different grape varieties.

*Vitis vinifera* × *Vitis labrusca* cv. ‘Jumeigui’, a popular hybrid cultivar, is highly valued for its intense muscat flavor and appealing appearance [18]. Nonetheless, its cultivation is challenged by issues such as low natural fruit set, the presence of seeds, and inconsistent berry size, which directly impact yield and marketability. Current research on PGRs in grapes has provided insights into various cultivars. For example, studies on ‘Hongyan Wuhe’ have shown that treatments with GA_3_ and TDZ can improve fruit quality, with GA_3_ alone sometimes yielding the best comprehensive results [19]. Research on ‘Red Globe’ indicates that combinations of GA_3_ with CPPU or 6-BA can effectively promote berry growth [17,20]. However, there is a paucity of systematic research focusing specifically on the ‘Jumeigui’ cultivar. It remains unclear how varying concentrations of CPPU, TDZ, and 6-BA in combination with GA_3_ differentially affect seedless-fruit induction. Their comparative impacts on subsequent external and internal fruit quality also represent a critical knowledge gap.

Therefore, the objectives of this study were: (1) to investigate and compare the effects of different concentrations of CPPU, TDZ, and 6-BA in combination with GA_3_ on seedless rate and berry drop (assessed via fruit stem brush length) in ‘Jumeigui’ grapes; (2) to comprehensively evaluate the impacts of these treatments on key fruit quality parameters. The findings aim to identify an optimal, quality-oriented PGR protocol for ‘Jumeigui’ grape seedless production, providing a scientific basis for growers to achieve high yields of premium fruit.

## 2. Materials and Methods

### 2.1. Plant Material

The test material consisted of four-year-old ‘Jumeigui’ grapevines. The rows were oriented north–south with a plant spacing of 1.0 m × 2.0 m (plant × row). The vines were trained on a V-shaped trellis system and cultivated in a plastic greenhouse to avoid rain. All other cultivation management and pest control measures followed conventional methods.

### 2.2. Experimental Design and Treatments

The seedless fruit-setting treatment was applied to ‘Jumeigui’ grapes at 2 days after flowering (on 14 April 2025) according to the following experimental design scheme (Table 1): ① CK (blank control); ② A (18 mg/L GA_3_ + 0.5 mg/L CPPU); ③ B (18 mg/L GA_3_ + 1 mg/L CPPU); ④ C (18 mg/L GA_3_ + 1.5 mg/L CPPU); ⑤ D (18 mg/L GA_3_ + 0.5 mg/L TDZ); ⑥ E (18 mg/L GA_3_ + 1 mg/L TDZ); ⑦ F (18 mg/L GA_3_ + 1.5 mg/L TDZ); ⑧ G (18 mg/L GA_3_ + 10 mg/L 6-BA); ⑨ H (18 mg/L GA_3_ + 20 mg/L 6-BA); ⑩ I (18 mg/L GA_3_ + 30 mg/L 6-BA). A uniform treatment of 25 mg/L GA_3_ + 30 mg/L 6-BA was used for fruit enlargement (on 26 April 2025) to investigate the effects of different seedless treatment combinations on the growth and fruit quality of ‘Jumeigui’ grapes. Three plants constituted one plot, with each treatment replicated three times, resulting in a total of nine plants per treatment. There were ten treatments in total, with a grand total of 90 grapevines as samples.

### 2.3. Measurement of Fruit Quality Indices

#### 2.3.1. Fruit Appearance Indices

1. Bunch length, bunch weight, and berry weight, and other related indices: Bunch length was measured using a straight ruler. Bunch weight and berry weight were determined using an electronic balance (LT502E, Changshu Tianliang Instrument Co., Ltd., Suzhou, China), which was accurate to 0.01 g. Single-berry weight was calculated as the total weight divided by the number of berries. Berry longitudinal and transverse diameters were measured with a vernier caliper (ARZ-1331, Eirezer AG, Qingdao, China), accurate to 0.01 cm, and the fruit shape index was calculated as the ratio of longitudinal to transverse diameter. The length of the fruit stalk brush was also measured with a vernier caliper. For each treatment, 30 berries were measured, and the average was taken. For seedless rate determination, 30 randomly selected berries per treatment were cut along the equator and examined. This was repeated three times, and the average value was calculated.
2. Bunch coloration grade: The bunch coloration grade was assessed and averaged according to the survey criteria established by Li et al. [21] (Table 2).
3. Color Index of Red Grape (CIRG): A CR-400 handheld colorimeter (Konica Minolta, Tokyo, Japan) was used to measure the color indices L*, a*, and b* at the equatorial region of the fruit. For each treatment, 30 berries were measured, and the average was calculated. The CIRG was then computed based on L*, a*, and b* values [21]. The CIRG scale for evaluating fruit appearance color is as follows: CIRG < 2 indicates yellow-green, 2 < CIRG < 4 indicates pink, 4 < CIRG < 5 indicates red, 5 < CIRG < 6 indicates dark red, and CIRG > 6 indicates blue-black [22].

#### 2.3.2. Fruit Internal Indices (Physicochemical Indices)

1. Soluble solids content (SSC) and titratable acidity (TA): Soluble solids content was measured using a refractometer (ATAGO PAL-1, Atago Co., Ltd., Tokyo, Japan). Titratable acidity was determined using an acid meter (PAL-Easy ACID2, Atago Co., Ltd., Tokyo, Japan). The solid–acid ratio was calculated as the ratio of soluble solids content to titratable acidity.
2. Ascorbic acid, tannin, bitterness/astringency, and anthocyanin contents: The ascorbic acid content in grapes was determined using the spectrophotometric method, which was based on the chemical reducibility of ascorbic acid and its reaction with specific reagents to form a colored compound. This compound absorbs light at a specific wavelength, and its absorbance is directly proportional to its concentration [23]. Tannin content was measured according to the Chinese agricultural industry standard NY/T 1600-2008 ‘Determination of tannin content in fruit, vegetable and derived product-Spectrophotometry method.’ [24]. The fundamental principle of this method was as follows: tannin substances, under alkaline conditions, could reduce tungstomolybdic acid to produce a blue-colored complex. This blue complex exhibited maximum absorption at a wavelength of 765 nm. Within a certain concentration range, the absorbance value was directly proportional to the tannin content (calculated as gallic acid), allowing for quantitative analysis. Bitterness and astringency were assessed using an electronic tongue (INSENT SA402B, Insent Co., Ltd., Tokyo, Japan). Anthocyanin content in the grape skin was determined using the pH differential method, which was based on the reversible structural transformation of anthocyanin molecules in response to changes in solution pH, which systematically altered their maximum absorption wavelength and absorbance. By measuring the absorbance of the sample extract at specific wavelengths under two buffered systems (pH 1.0 and pH 4.5), and calculating the differential absorbance, interference from non-anthocyanin pigments was eliminated, allowing for the specific quantification of anthocyanins.

### 2.4. Statistical and Multivariate Analysis

The experimental data were subjected to comprehensive statistical analysis using Microsoft Excel 2019 for preliminary data organization and Origin 2018 for graphical representations, and one-way ANOVA with Duncan’s multiple comparisons was performed using https://www.spsspro.com (on 14 November 2025) for advanced statistical computations at a significance level of 0.05. The data were presented as mean ± standard deviation (SD). Principal component analysis (PCA) was performed using https://www.spsspro.com, and the data were standardized prior to analysis to eliminate scale differences among variables.

## 3. Results

### 3.1. Appearance Quality Parameters

**Figure 1:**
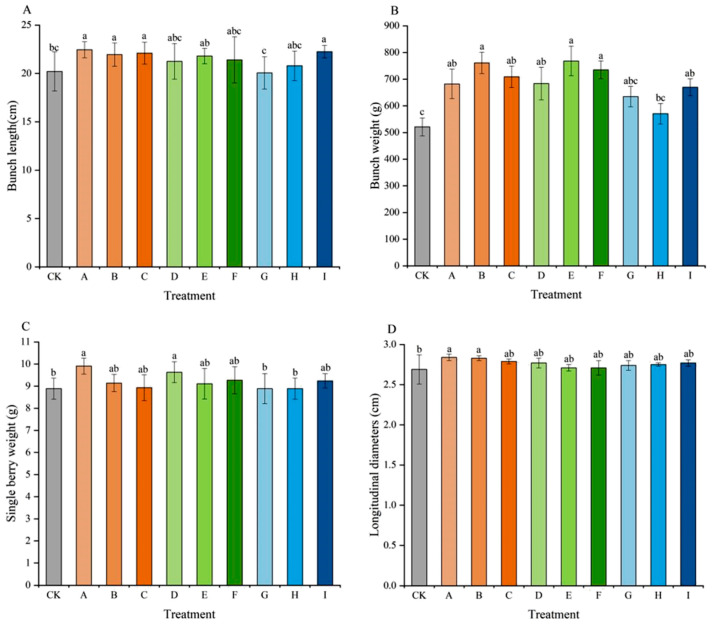

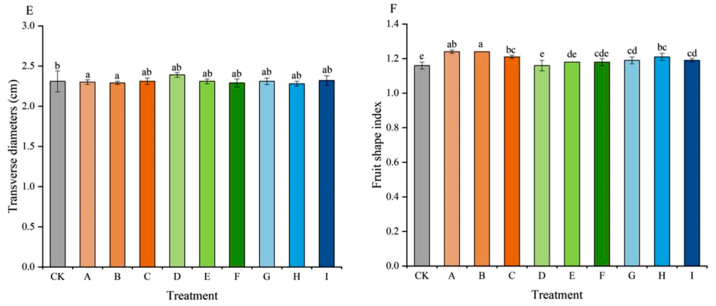
Effects of different treatments on fruit bunch length (**A**), bunch weight (**B**), single-berry weight (**C**), longitudinal diameter (**D**), transverse diameter (**E**) and fruit shape index (**F**) of *Vitis vinifera* × *Vitis labrusca* cv. ‘Jumeigui’. Different lowercase letters indicated significant differences according to Duncan’s multiple range test (*p* < 0.05). CK: control, A: 18 mg/L GA_3_ + 0.5 mg/L CPPU, B: 18 mg/L GA_3_ + 1 mg/L CPPU, C: 18 mg/L GA_3_ + 1.5 mg/L CPPU, D: 18 mg/L GA_3_ + 0.5 mg/L TDZ, E: 18 mg/L GA_3_ + 1 mg/L TDZ, F: 18 mg/L GA_3_ + 1.5 mg/L TDZ, G: 18 mg/L GA_3_ + 10 mg/L 6-BA, H: 18 mg/L GA_3_ + 20 mg/L 6-BA, I: 18 mg/L GA_3_ + 30 mg/L 6-BA.

The results of this study indicated that different seedless treatments exerted distinct effects on the external traits of grape berries (Figure 1). Regarding bunch length (Figure 1A), treatments A, B, C, and I were all significantly higher than the control (CK), with treatment A achieving the maximum length of 22.45 cm. No significant differences were observed between the remaining treatments and CK. The bunch weight for all treatments exceeded that of CK (521.25 g) (Figure 1B). Treatments E and B showed relatively higher bunch weights, increasing by 47.39% and 46.01% compared to CK, respectively. For single-berry weight (Figure 1C), treatment A exhibited a relatively larger berry weight (9.91 g), followed by treatment D (9.63 g), and they were both significantly higher than that of CK. Regarding fruit morphology, the longitudinal diameter of all treatments was greater than that of CK (2.69 cm) (Figure 1D). Treatments A and B were relatively larger, reaching 2.84 cm and 2.83 cm, respectively, which were significantly greater than those of CK. The differences between the other treatments and CK were not significant. The maximum transverse diameter was observed in treatment D (2.39 cm) (Figure 1E), while the other treatments ranged between 2.28 cm and 2.32 cm, showing minor differences. The fruit shape index was higher for treatments A and B (both 1.24) (Figure 1F), indicating a shape closer to an elongated round. The values for the remaining treatments ranged from 1.16 to 1.21, corresponding to a shape more typical of a standard ellipse.

**Figure 2:**
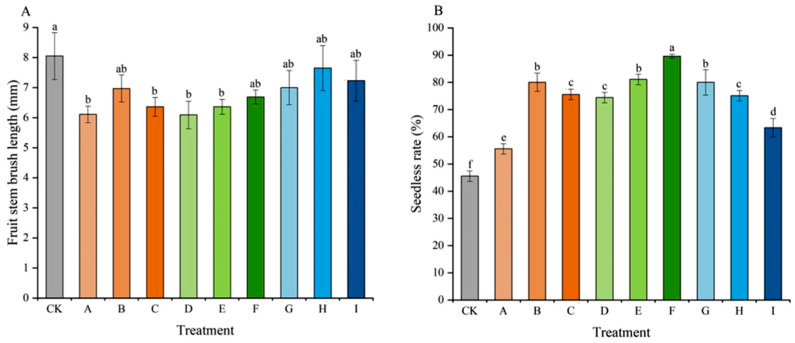
Effects of different treatments on fruit stem brush length (**A**) and seedless rate (**B**) of ‘Jumeigui’ grapes. Different lowercase letters indicated significant differences according to Duncan’s multiple range test (*p* < 0.05). CK: control, A: 18 mg/L GA_3_ + 0.5 mg/L CPPU, B: 18 mg/L GA_3_ + 1 mg/L CPPU, C: 18 mg/L GA_3_ + 1.5 mg/L CPPU, D: 18 mg/L GA_3_ + 0.5 mg/L TDZ, E: 18 mg/L GA_3_ + 1 mg/L TDZ, F: 18 mg/L GA_3_ + 1.5 mg/L TDZ, G: 18 mg/L GA_3_ + 10 mg/L 6-BA, H: 18 mg/L GA_3_ + 20 mg/L 6-BA, I: 18 mg/L GA_3_ + 30 mg/L 6-BA.

The fruit stem brush length for all treatments was shorter than that of the CK (Figure 2A), which indicated that the seedless treatments utilizing CPPU, TDZ, and 6-BA differentially influenced the berry abscission potential of ‘Jumeigui’ grapes. Among the treatments, G, H, and I exhibited relatively longer brush lengths, showing no significant difference from the CK. This result suggests that the application of GA_3_ + 6-BA for seedless-fruit setting rendered the berry clusters of ‘Jumeigui’ grapes less susceptible to shedding. The seedless rate was significantly enhanced in all treatments compared to the CK (45.56%) (Figure 2B). Treatment F yielded the highest seedless rate at 89.56%, followed by treatments E, B, and G, which also showed rates above 85%. In this study, elevated concentrations of CPPU and TDZ effectively promoted seedless fruit formation in ‘Jumeigui’ grapes, whereas 6-BA exhibited an opposing trend.

### 3.2. Internal Quality Parameters

**Figure 3:**
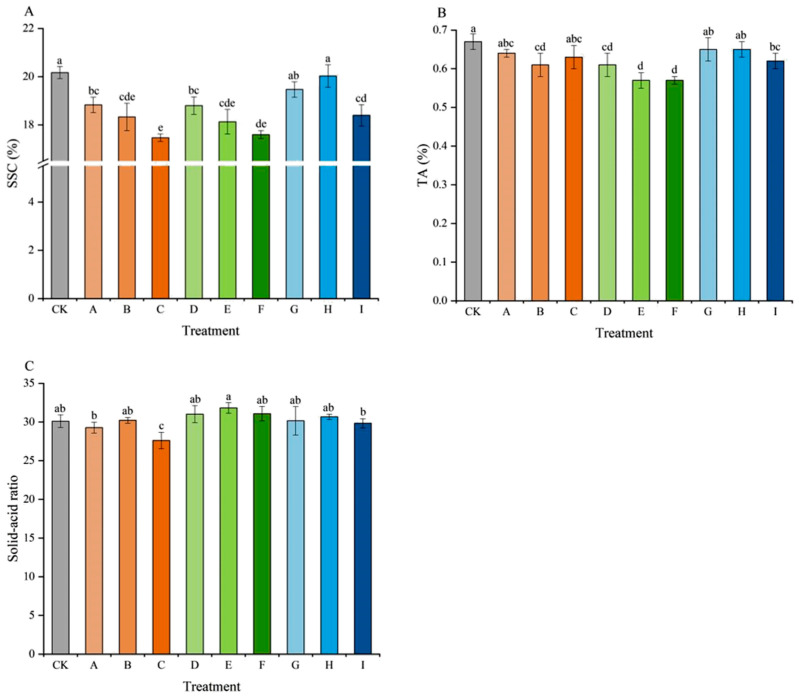
Effects of different treatments on SSC (**A**), TA (**B**), and solid–acid ratio (**C**) of ‘Jumeigui’ grapes. Different lowercase letters indicated significant differences according to Duncan’s multiple range test (*p* < 0.05). CK: control, A: 18 mg/L GA_3_ + 0.5 mg/L CPPU, B: 18 mg/L GA_3_ + 1 mg/L CPPU, C: 18 mg/L GA_3_ + 1.5 mg/L CPPU, D: 18 mg/L GA_3_ + 0.5 mg/L TDZ, E: 18 mg/L GA_3_ + 1 mg/L TDZ, F: 18 mg/L GA_3_ + 1.5 mg/L TDZ, G: 18 mg/L GA_3_ + 10 mg/L 6-BA, H: 18 mg/L GA_3_ + 20 mg/L 6-BA, I: 18 mg/L GA_3_ + 30 mg/L 6-BA.

As shown in Figure 3, this study compared the effects of different seedless treatments on the internal nutritional quality of grape berries. The specific results were as follows: The soluble solids content (SSC) was higher in the CK and treatment H (Figure 3A), reaching 20.17% and 20.03%, respectively, while treatment C had the lowest SSC (17.47%). The SSC level gradually decreased with increasing concentrations of CPPU and TDZ. Among the 6-BA treatments, 18 mg/L GA_3_ + 20 mg/L 6-BA (treatment H) yielded the highest SSC. The titratable acidity (TA) of all treatments was lower than that of the CK (0.67%) (Figure 3B). Treatments E and F had the lowest TA (0.57%), which was significantly lower than that of the CK. The solid–acid ratio was the lowest in treatment C (27.61) (Figure 3C), indicating poorer flavor balance. In contrast, treatments D, E, and F exhibited relatively higher solid–acid ratios, suggesting that grapes treated with GA_3_ + TDZ possessed better flavor. Among them, 18 mg/L GA_3_ + 1 mg/L TDZ (treatment E) achieved the highest solid–acid ratio (31.82), representing the optimal flavor balance.

**Figure 4:**
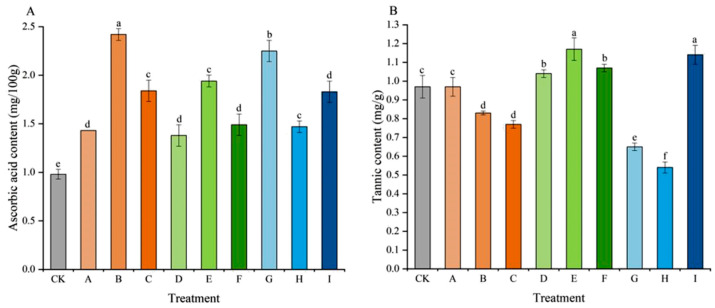
Effects of different treatments on ascorbic acid content (**A**) and tannin content (**B**) of ‘Jumeigui’ grapes. Different lowercase letters indicated significant differences according to Duncan’s multiple range test (*p* < 0.05). CK: control, A: 18 mg/L GA_3_ + 0.5 mg/L CPPU, B: 18 mg/L GA_3_ + 1 mg/L CPPU, C: 18 mg/L GA_3_ + 1.5 mg/L CPPU, D: 18 mg/L GA_3_ + 0.5 mg/L TDZ, E: 18 mg/L GA_3_ + 1 mg/L TDZ, F: 18 mg/L GA_3_ + 1.5 mg/L TDZ, G: 18 mg/L GA_3_ + 10 mg/L 6-BA, H: 18 mg/L GA_3_ + 20 mg/L 6-BA, I: 18 mg/L GA_3_ + 30 mg/L 6-BA.

For ascorbic acid content (Figure 4A), all treatments were significantly higher than the CK (0.98 mg/100 g). Treatment B showed the highest ascorbic acid content at 2.42 mg/100 g, followed by treatment G (2.25 mg/100 g), representing increases of 146.94% and 129.59% over the CK, respectively. Within the CPPU and TDZ treatment groups, the highest ascorbic acid contents were observed in 18 mg/L GA_3_ + 1 mg/L CPPU (treatment B) and 18 mg/L GA_3_ + 1 mg/L TDZ (treatment E). The tannin content was relatively higher in treatments D, E, F, and I (Figure 4B), ranging between 1.04 and 1.17 mg/g. In contrast, treatment H exhibited the lowest tannin content (0.54 mg/g), followed by treatment G, which suggested that low concentrations of 6-BA could reduce the tannin content in ‘Jumeigui’ grapes, thereby decreasing astringency.

### 3.3. Fruit Coloring Parameters

**Figure 5:**
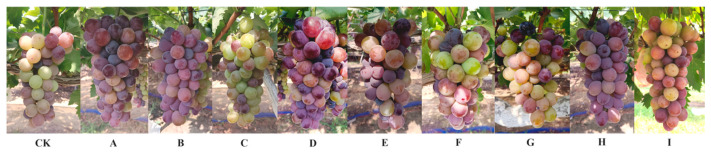
The status of ‘Jumeigui’ grapes at maturity under different seedless treatments. CK: control, A: 18 mg/L GA_3_ + 0.5 mg/L CPPU, B: 18 mg/L GA_3_ + 1 mg/L CPPU, C: 18 mg/L GA_3_ + 1.5 mg/L CPPU, D: 18 mg/L GA_3_ + 0.5 mg/L TDZ, E: 18 mg/L GA_3_ + 1 mg/L TDZ, F: 18 mg/L GA_3_ + 1.5 mg/L TDZ, G: 18 mg/L GA_3_ + 10 mg/L 6-BA, H: 18 mg/L GA_3_ + 20 mg/L 6-BA, I: 18 mg/L GA_3_ + 30 mg/L 6-BA.

There were significant differences in the maturity status of ‘Jumeigui’ grape under different seedless berry treatments, as shown in Figure 5.

**Figure 6:**
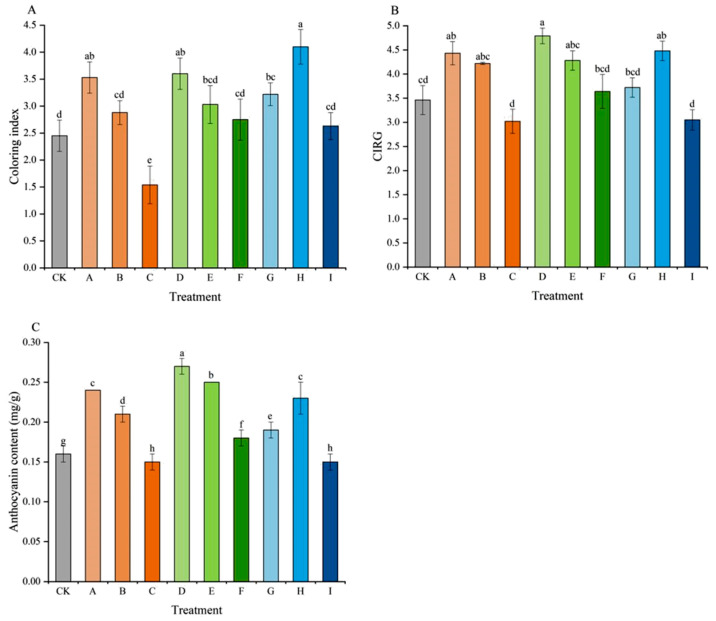
Effects of different treatments on coloring index (**A**), CIRG (**B**), and anthocyanin content (**C**) of ‘Jumeigui’ grapes. Different lowercase letters indicated significant differences according to Duncan’s multiple range test (*p* < 0.05). CK: control, A: 18 mg/L GA_3_ + 0.5 mg/L CPPU, B: 18 mg/L GA_3_ + 1 mg/L CPPU, C: 18 mg/L GA_3_ + 1.5 mg/L CPPU, D: 18 mg/L GA_3_ + 0.5 mg/L TDZ, E: 18 mg/L GA_3_ + 1 mg/L TDZ, F: 18 mg/L GA_3_ + 1.5 mg/L TDZ, G: 18 mg/L GA_3_ + 10 mg/L 6-BA, H: 18 mg/L GA_3_ + 20 mg/L 6-BA, I: 18 mg/L GA_3_ + 30 mg/L 6-BA.

In terms of fruit coloring index, CIRG, and anthocyanin accumulation, differences were also observed among the treatments (Figure 6). Treatment H exhibited the highest coloring index (Figure 6A), followed by treatments D and A, while treatments C and I showed relatively lower indices. Regarding the CIRG value (Figure 6B), treatment D (4.79) ranked highest again, followed by treatment H (4.48) and treatment A (4.43), which were significantly higher than the CK (3.46). Treatments C and I had the lowest CIRG values, at 3.02 and 3.05, respectively.

For anthocyanin content (Figure 6C), treatment D performed best, reaching 0.27 mg/g, followed by treatments E (0.25 mg/g) and A (0.24 mg/g). In contrast, treatments C and I had the lowest anthocyanin contents, both at 0.15 mg/g, which were significantly lower than those of other treatments. In this study, all treatments exhibited the same trend for coloring index, CIRG, and anthocyanin accumulation: the coloring effect gradually decreased with increasing concentrations of CPPU and TDZ, while it initially increased and then decreased with increasing 6-BA concentration.

### 3.4. Comprehensive Evaluation of Different Treatments

Principal component analysis (PCA) was conducted on the comprehensive indicators of ‘Jumeigui’ grapes subjected to different treatments (Table 3). Four principal components were extracted, with a cumulative contribution rate of 92.135%, effectively reflecting the overall effects of the various treatments on ‘Jumeigui’ grapes. The first principal component (PC1), with a contribution rate of 33.865%, encompassed the original information of four traits: fruit stem brush length, titratable acidity (TA), CIRG, and anthocyanin content. The second principal component (PC2), contributing 29.802%, represented the original information of two traits: soluble solids content (SSC) and coloring index. The third principal component (PC3), with a contribution rate of 17.883%, accounted for the original information of four traits: single-berry weight, seedless rate, ascorbic acid content, and tannin content. The fourth principal component (PC4), contributing 10.585%, represented the original information of one trait: the solid–acid ratio.

**Figure 7:**
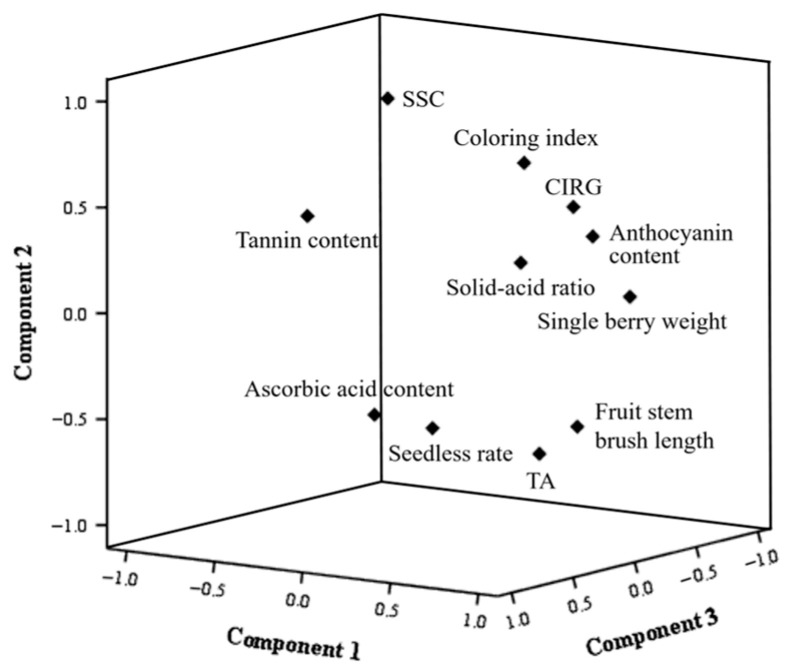
3-D Component Plot in Rotated Factor Space.

The factor-loading diagram reduced multiple factors into three principal components and presented the spatial distribution of the principal components through a 3-D Component Plot in Rotated Factor Space (Figure 7). A strong correlation was observed between SSC, coloring index, CIRG, anthocyanin content, solid–acid ratio and single-berry weight, as well as between fruit stem brush length, TA, seedless rate, and ascorbic acid content.

The PCA comprehensive score is represented by the Z-value, where a higher value indicates a better overall effect. The comprehensive evaluation results were ranked as H > D > E > A > G > B > F > CK > I > C (Table 4). Treatment H achieved the highest score, indicating that it delivered the best overall effect. The findings of this study demonstrate that the seedless treatment with 18 mg/L GA_3_ + 20 mg/L 6-BA yielded the most significant improvement in the overall quality of ‘Jumeigui’ grapes.

## 4. Discussion

Improving fruit quality and commodity value while conducting seedless induction is the most important goal in the planting process of ‘Jumeigui’ grape. This study provided a comprehensive analysis of how different cytokinins, in combination with GA_3_, affected fruit seedless induction and the resulting quality profile of ‘Jumeigui’ grapes.

Previous studies showed that CPPU, TDZ, or 6-BA could increase berry size and cluster weight [8,12,15]. In this study, all treatments promoted the enlargement of berries compared with the CK. Low concentrations (0.5 mg/L) of CPPU or TDZ in combination with GA_3_ could significantly increase berry size while higher concentrations result in smaller berries. In contrast, 6-BA exhibited the opposite trend, with a high concentration (30 mg/L) significantly promoting berry enlargement. This supported the observation that different PGRs had varying efficiencies in promoting cell division versus cell expansion. Furthermore, evaluation of the fruit shape index revealed that berries treated with TDZ were elongated and round, whereas those treated with CPPU or 6-BA were elliptical (Figure 1F), which was consistent with a previous study [25].

A primary objective of seedless treatment was to maximize seedless rates. Under normal circumstances, ‘Jumeigui’ grapes could undergo parthenocarpy, leading to seedless berries, but these berries were generally smaller, causing the entire cluster to exhibit uneven fruit size and irregular berry uniformity. The use of growth regulators for seedless treatment could make grape clusters more uniform and aesthetically pleasing while achieving seedlessness. Our data aligned with established research [26,27], confirming that within a certain concentration range, high concentrations of CPPU and TDZ were highly effective for this purpose, with treatment F (1.5 mg/L TDZ) achieving the highest rate of 89.56%. However, this high efficacy came at a cost. Treatments with CPPU and TDZ significantly shortened the fruit stem brush length, a direct indicator of increased abscission layer development and higher susceptibility to berry drop. This finding was critical for post-harvest handling and marketability, as berries with fragile pedicels were prone to shattering during transport. In contrast, treatments with 6-BA showed fruit stem brush lengths comparable to the CK, indicating that berry attachment remained robust. This suggested that 6-BA may regulate abscission-related hormones differently or induce less stress on the pedicel zone compared to CPPU and TDZ.

SSC and TA were important indicators for grape flavor evaluation. The results of Kok et al. (2016) [28] indicated that the application of TDZ reduced the SSC of ‘Recel Uzümü’ grapes; with increasing concentration, SSC gradually decreased. The same results were found in ‘Campbell Early’ [29]. The use of CPPU also reduced the SSC of ‘Flame Seedless’ grapes [30]. In this study, the results showed that SSC significantly declined with increasing CPPU and TDZ concentration (with treatment C being the lowest), which was consistent with previous studies. These regulators seemed to delay ripening or create a metabolic “sink” competition, diverting resources towards growth rather than sugar accumulation. 6-BA was associated with the altered expression of multiple genes involved in starch and sucrose metabolism and cellular polysaccharide metabolic processes [31]. Therefore, in this study, the application of appropriate concentrations of 6-BA (10 and 20 mg/L) for seedless induction in ‘Jumeigui’ grapes might not lead to a reduction in soluble solids content, which was consistent with previous findings on other cultivars suggesting a milder impact of 6-BA on carbohydrate metabolism [32]. The superior solid–acid ratio in TDZ treatments (especially E) indicated a favorable shift in flavor balance, primarily driven by a more pronounced reduction in titratable acidity. This suggested that TDZ might hasten acid degradation or metabolism more effectively than other regulators during the study period.

Ascorbic acid is an important antioxidant that plays crucial roles in cancer prevention, enhancing human immunity, and improving stress response capacity [19]. Ascorbic acid participates in redox metabolism in plant tissues, thereby enhancing the plant organism’s resistance to infections and low temperatures [33]. A notable positive finding in this study was the sharp and uniform increase in ascorbic acid content across all PGR treatments. This presents a potential avenue for future research, requiring further investigation including the analysis of cytokinin signaling gene expression and hormone quantification to validate this hypothesis. The astringent taste of fruit primarily comes from tannins [19]. Lower-concentration 6-BA treatments (G, H) in this study reduced tannin content while TDZ treatments generally increased tannin content in grapes, leading to enhanced astringency, which may be related to the flavonoid metabolic pathway [34].

Fruit color was also an important index of fruit quality [35]. The results of this study clearly demonstrated an inhibitory effect of high-concentration CPPU and TDZ on skin pigmentation, as seen in the low CIRG and anthocyanin content of treatments C and I. This inhibition was likely linked to delayed physiological ripening and potentially to a cytokinin-mediated down-regulation of genes in the anthocyanin pathway [29]. Conversely, treatments A (0.5 mg/L CPPU), D (0.5 mg/L TDZ), and H (20 mg/L 6-BA) excelled in promoting coloration. The mechanism for CPPU and TDZ might be concentration-dependent, with low doses potentially acting as a ripening stimulant. A previous study indicated that 6-BA treatment induced phenylalanine ammonia lyase (PAL) activity, which was favorable to the synthesis of anthocyanin and maintenance of anthocyanin content [36]. In this study, the positive effect of an optimal 6-BA concentration (20 mg/L) was particularly noteworthy, indicating its potential to enhance quality aspects without the strong ripening suppression associated with other cytokinins.

For a quality-oriented premium market, where flavor and color are paramount, 18 mg/L GA_3_ combined with 20 mg/L 6-BA emerges as a compelling strategy. This combination balanced a high seedless rate with excellent SSC, the best coloration index, and minimal impact on berry attachment. This approach may be especially valuable for ‘Jumeigui’ grapes, a cultivar whose signature rose fragrance and soft fruit texture require careful management.

Recognizing the moderate sample size of this study as a potential limitation, it was suggested that future studies involve larger-scale trials for validation.

## 5. Conclusions

This study systematically evaluated the impact of different seedless treatments on multiple quality parameters of ‘Jumeigui’ grapes. Overall, as the concentrations of CPPU and TDZ increased, the comprehensive effect on ‘Jumeigui’ grapes gradually declined. Seedless treatments using 18 mg/L GA_3_ + 0.5 mg/L CPPU or TDZ demonstrated relatively good improvements in the overall quality of the grapes. In contrast, as the concentration of 6-BA increased, the comprehensive effect first improved and then declined. Among all treatments, 18 mg/L GA_3_ + 20 mg/L 6-BA provided the most significant enhancement in the overall quality, which is of great significance for the commercial cultivation of ‘Jumeigui’ grapes.

## Figures and Tables

**Table 1:** Experimental design scheme of different seedless fruit treatments of *Vitis vinifera* × *Vitis labrusca* cv. ‘Jumeigui’.

Treatments	Seedless Fruit-Setting Treatment (on 14 April 2025)	Fruit Enlargement Treatment (on 26 April 2025)
CK (blank control)	--	25 mg/L GA_3_ + 30 mg/L 6-BA
A	18 mg/L GA_3_ + 0.5 mg/L CPPU	
B	18 mg/L GA_3_ + 1 mg/L CPPU	
C	18 mg/L GA_3_ + 1.5 mg/L CPPU	
D	18 mg/L GA_3_ + 0.5 mg/L TDZ	
E	18 mg/L GA_3_ + 1 mg/L TDZ	
F	18 mg/L GA_3_ + 1.5 mg/L TDZ	
G	18 mg/L GA_3_ + 10 mg/L 6-BA	
H	18 mg/L GA_3_ + 20 mg/L 6-BA	
I	18 mg/L GA_3_ + 30 mg/L 6-BA	

**Table 2:** Standards for the coloration grade of the bunch.

Class	The Percentage of Coloring Area to the Whole Bunch
1	Coloring area ≤ 30%
2	30% < Coloring area ≤ 50%
3	50% < Coloring area ≤ 70%
4	70% < Coloring area ≤ 90%
5	Coloring area > 90%

**Table 3:** Principal component analysis of fruit quality evaluation factors of ‘Jumeigui’ grape.

Principal Component	PC1	PC2	PC3	PC4
Eigen value	3.725	3.278	1.967	1.164
Contribution ratio (%)	33.865	29.802	17.883	10.585
Cumulative contribution ratio (%)	33.865	63.668	81.55	92.135
Single-berry weight	0.618	−0.003	−0.666 *	0.274
Fruit stem brush length	0.619 *	−0.555	−0.237	0.416
Seedless rate	0.463	−0.440	0.717 *	0.004
SSC	−0.270	0.944 *	0.034	−0.095
TA	0.666 *	−0.625	0.138	−0.359
Solid–acid ratio	0.633	0.290	0.243	−0.667 *
Ascorbic acid content	0.091	−0.423	0.656 *	0.287
Tannin content	−0.295	0.475	0.649 *	0.441
Coloring index	0.587	0.743 *	0.149	0.071
CIRG	0.794 *	0.541	0.045	0.156
Anthocyanin content	0.871 *	0.402	−0.002	0.166

Note: * means the biggest absolute value of each index in all factors.

**Table 4:** The comprehensive evaluation of the effects of different treatments.

Treatments	CK	A	B	C	D	E	F	G	H	I
Z value	−0.969	0.471	0.268	−1.589	1.096	0.542	−0.353	0.313	1.319	−1.098
Rank	8	4	6	10	2	3	7	5	1	9

## Data Availability

The data presented in this study are available on request from the corresponding author.

## References

[1] Varoquaux F., Blanvillain R., Delseny M., Gallois P. (2000). Less is better: New approaches for seedless fruit production. Trends Biotechnol..

[2] Wang S., Cheng D.W., Li M., Gu H., Li Z.Y., Qi S., Chen J.Y. (2020). Research Progress on Seedless-Induced Production of ‘Shine Muscat’ Grape. J. Agric. Sci. Technol..

[3] Li X., Cai Z., Liu X., Wu Y., Han Z., Yang G., Li S., Xie Z., Liu L., Li B. (2024). Effects of Gibberellic Acid on Soluble Sugar Content, Organic Acid Composition, Endogenous Hormone Levels, and Carbon Sink Strength in Shine Muscat Grapes during Berry Development Stage. Horticulturae.

[4] Ishikawa H., Togano Y., Shibuya T. (2024). Profile of gene expression at the berry enlargement phase of the large berry mutant of ‘Delaware’ grape. Acta Hortic..

[5] Alshallash K.S., Fahmy M.A., Tawfeeq A.M., Baghdady G.A., Abdrabboh G.A., Hamdy A.E., Kabsha E.-b.A. (2023). GA_3_ and Hand Thinning Improves Physical, Chemical Characteristics, Yield and Decrease Bunch Compactness of Sultanina Grapevines (*Vitis vinifera* L.). Horticulturae.

[6] Dong J., Zhang P., Li W., Li H., Zhou G., Chen K., Fang Y., Zhang K. (2025). Effects of Seedlessness and Swelling Treatments Based on GA_3_ and CPPU on the Fruit Quality of “Shine Muscat” Grapes. Sci. Agric. Sin..

[7] Hassan A.E., Behary E.H.M. (2021). Effect of some gibberellic acid and forchlorfnuron application on productivity and berries development of early sweet grapes. Menoufia J. Plant Prod..

[8] Han X., Mi Y., Wang H., Ye S., Abe-Kanoh N., Ji W. (2025). Influence of GA_3_ and CPPU on the Quality Attributes and Peelability of ‘Wuhe Cuibao’ Grape. Agronomy.

[9] Peng T., Liu C., Wu S.L., Li J.B., Liu F. (2020). Effects of different puffing measures on the fruit quality of ‘summer black’ grapes. IOP Conf. Ser. Earth Environ. Sci..

[10] Rademacher W. (2015). Plant Growth Regulators: Backgrounds and Uses in Plant Production. J. Plant Growth Regul..

[11] Peppi M.C., Fidelibus M.W. (2008). Effects of Forchlorfenuron and Abscisic Acid on the Quality of ‘Flame Seedless’ Grapes. HortScience.

[12] Ren J., Li X., Song X., Ren C., Shen Y., Tao J. (2013). Effects of GA_3_ and TDZ on Fruit Growth and Quality of Summer Black Grape. Acta Agric. Jiangxi.

[13] Yang P., Wu Z., Liu B., Wang L., Wang S. (2025). Synergistic Effects of Gibberellic Acid, Forchlorfenuron, Thidiazuron, and Brassinosteroid Combinations on Seedless Berry Development and Quality Enhancement in ‘Shine Muscat’ and ‘Red Muscat of Alexandria’ Grapes. Biology.

[14] Lee S.Y., Heo J.Y. (2023). Combined treatment with gibberellic acid and thidiazuron improves fruit quality of ‘Red Dream’ grape cultivar. Not. Sci. Biol..

[15] Choi S., Ban S., Choi C. (2023). The Impact of Plant Growth Regulators and Floral Cluster Thinning on the Fruit Quality of ‘Shine Muscat’ Grape. Horticulturae.

[16] Huo S.S., Xi Z.M., Ma L.N., Luan L.Y., Luan L.Y. (2012). Effect of plant growth regulator on the quality of Cabernet sauvignon grape. J. Northwest A F Univ.—Nat. Sci. Ed..

[17] He J., Guo C.B., Wang P., Zheng Q.Q., Zhao L., Wu M., Lei Y.J. (2012). Effects of plant growth regulator on the fruit quality of Red Globe. Sino-Overseas Grapevine Wine.

[18] Leng X.P., Cong J.M., Cheng L.X., Wan H.L., Liu Y.X., Yuan Y.B., Fang J.G. (2023). Identification of key gene networks controlling monoterpene biosynthesis during grape ripening by integrating transcriptome and metabolite profiling. Hortic. Plant J..

[19] Cheng D.W., He S.S., Gu S.C., Li M., Guo X.Z., Gu H., Chen J.Y. (2021). Influence of GA_3_ and TDZ on fruit quality of ‘Hongyan Wuhe’ grape. J. Fruit Sci..

[20] Strydom J. (2013). Research Note: Effect of CPPU (*N*-(2-Chloro-4-Pyridinyl)-*N’*-Phenylurea) and a Seaweed Extract on Flame Seedless, Redglobe and Crimson Seedless Grape Quality. S. Afr. J. Enol. Vitic..

[21] Li F.F., Wang S., Gu S.C., Cheng D.W., Gu H., Li M., Chen J.Y., Yang Y.J. (2020). Effects of foliar application of ABA and PDJ on the coloration and quality of ‘Kyoho’ grape berry. J. Fruit Sci..

[22] Amiri M.E., Fallahi E., Parseh S.H. (2010). Application of ethephon and ABA at 40% veraison advanced maturity and quality of ‘Beidaneh Ghermez’ grape. Xi Int. Symp. Plant Bioregul. Fruit Prod..

[23] Wang H.F., Shao X.F. (2012). Experimental Guide for Fruit and Vegetable Storage and Processing.

[24] (2008). Determination of Tannin Content in Fruit, Vegetable and Derived Product—Spectrophotometry Method.

[25] Shin H.W., Kim G.H., Choi C. (2019). Effects of Plant Growth Regulators and Floral Cluster Thinning on Fruit Quality of ‘Shine Muscat’ Grape. Hortic. Sci. Technol..

[26] Wang J.P., Xu W.H., Zhang C., Song Z.Z., Cao Z.Y., Tang M.L. (2022). Effects of Different Plant Growth Regulators on Fruit Quality of ‘Sunshine Rose’. North. Hortic..

[27] Tan X.F. (2025). The Effects of Different Plant Growth Regulator Formulations on the Fruit Quality and Storability of ‘Shine Muscat’ Grapes. Master’s thesis.

[28] Kok D., Bal E. (2016). Seedless Berry Growth and Bioactive Compounds of cv. ‘Recel Uzümü’ (*V. vinifera* L.) as Affected by Application Doses and Times of Pre-Harvest Thidiazuron. Erwerbs-Obstbau.

[29] Kim I.L., Piao Y.L., Hwang Y.S., Lee J.C. (2002). Effects of synthetic cytokinin, thidiazuron on berry size and quality of ‘Campbell Early’ (*Vitis labruscana*) grapes. J. Korean Soc. Hortic. Sci..

[30] Khalil H.A. (2020). Improved Yield, Fruit Quality, and Shelf Life in ‘Flame Seedless’ Grapevine with Pre-Harvest Foliar Applications of Forchlorfenuron, Gibberellic Acid, and Abscisic Acid. J. Hortic. Res. Natl. Inst. Hortic. Res..

[31] Tang W., Yang C., Cao Y., Wang Z., Du P., Lin M. (2026). Exogenous 6-BA Inhibits Fruit Cracking by Regulating the Hormonal Balance and Transcriptome Characteristics of the Jujube Fruit Peel. Agronomy.

[32] Nie S.Q., Chen B., Liu K.Y., Shi X.H., Yang G.S., Zhong X.H., Xu F., Bai M. (2013). Effects of Foliage Spraying 6-BA on Leaf Senescence Physiology and Fruit Quality of Grape. Hunan Agric. Sci..

[33] Borodulina I.D., Vorotyntseva M.V., Makarova G.A., Zemtsova A.Y., Sokolova G.G. (2021). The Content of Vitamin C in the Grape Grown under the Conditions of Southwestern Siberia. Russ. J. Bioorg. Chem..

[34] Zheng T., Zhao P.C., Xiang J., Wei L.Z., Shen W.T., Wu J., Cheng J.H. (2024). Integrated transcriptomic and metabolomic analysis reveals the effects of forchlorfenuron and thidiazuron on flavonoid biosynthesis in table grape skins. Curr. Plant Biol..

[35] Hernanz D., Recamales Á.F., Meléndez-Martínez A.J., González-Miret M.L., Heredia F.J. (2008). Multivariate statistical analysis of the color-anthocyanin relationships in different soilless-grown strawberry genotypes. J. Agric. Food Chem..

[36] Zhang D.D., Xu X.F., Zhang Z.K., Jiang G.X., Feng L.Y., Duan X.W., Jiang Y.M. (2018). 6-Benzylaminopurine improves the quality of harvested litchi fruit. Postharvest Biol. Tec..

